# OPA1, a new mitochondrial target in cancer therapy

**DOI:** 10.18632/aging.104207

**Published:** 2020-11-13

**Authors:** Stephanie Herkenne, Luca Scorrano

**Affiliations:** 1 Department of Biology, University of Padova, Padova, Italy; 2 Veneto Institute of Molecular Medicine, Padova, Italy; 3 Interdisciplinary Cluster in Applied Genoproteomics (GIGA) Research Center, University of Liège, Liège, Belgium

**Keywords:** mitochondria, OPA1, cancer, angiogenesis

Cancer is one of the most prevalent diseases of the ageing western world population. It is now accepted that cancer is a complex disease resulting from the interplay between the tumor cell and its microenvironment. Cancer cells are highly proliferative, are protected from apoptosis and from the immune system and can transform their microenvironment. A key facet of cancer is the activation of quiescent blood vessels to induce neovascularization, to support tumor growth and metastasis dissemination [1]. Angiogenesis, the new blood vessel formation from preexisting one, is not only crucial for cancer, but also for embryogenesis, tissue repair and ovulation and is associated to inflammation and diabetic retinopathy. One of the yet unmet needs in anticancer therapy is how to abrogate its metastatization, often the primary cause of death for most parenchymatous tumors [2]. In this respect, antiangiogenic therapy that starves the tumor bulk from oxygen and nutrients and reduces the conduits for cancer cell dissemination outside of the primary tumor location is a widely explored avenue. Classically, antiangiogenic therapies inhibit Vascular Endothelial Growth Factor (VEGF), the main driver of angiogenesis. They comprise antagonist monoclonal antibodies like Bevacizumab, FDA/EMA approved for colon, lung, renal and breast cancer; or small molecules inhibitors of VEGF receptor tyrosine kinase activity like lapatinib, sunitinib, sorafenib, axitinib, and pazopanib, approved for breast, renal, soft tissues, hepatocellular and rarer cancers [3]. However, as these therapies are being used in the clinics, oncologists are confronted with the emergence of resistance, calling for the identification of new antiangiogenic therapies that do not directly target the VEGF pathway.

Unexpectedly, mitochondria may offer a solution here. In addition to their role as primary metabolic hubs of the cell, mitochondria orchestrate multiple signaling events and are key organelles in the execution of apoptosis. This multifaceted involvement in cell biology is controlled by the dynamic balance between mitochondrial fusion and fission and by inner mitochondrial membrane remodeling. A set of core players regulate fission (dynamin related protein 1 and its receptors mitochondrial fission factor, mitochondrial division 49 and 51, and fission 1) and fusion (mitofusin 1 and 2 and optic atrophy 1 -OPA1). OPA1 is essential for fusion and for the remodeling of the inner mitochondrial membrane, thereby regulating apoptosis, mitochondrial respiratory efficiency and cell proliferation, all hallmarks of cancer [4]. However, whether mitochondrial dynamics participates in angiogenesis and is a targetable component of cancer angiogenesis was unknown. By screening available databases of gene expression changes during angiogenesis we identified a rapid and specific OPA1 induction in response to angiogenic stimuli. In cellular, zebrafish and mouse models, OPA1 induction was required to modulate gene expression during angiogenesis. Indeed, when this angiogenic OPA1 induction is curtailed, uncontrolled activation of the transcription factor NFκB skews gene expression towards an anti-angiogenic profile. This ectopic NFκB activation is not due to changes in mitochondrial bioenergetics, but in calcium signaling, highlighting a further function of OPA1 in the control of mitochondrial calcium uptake [5]. OPA1 emerged also as a master regulator of lymphangiogenesis, i.e. the formation of new lymphatic vessels from endothelial cells. In the context of cancer, angiogenesis is used by the primary tumor to grow exponentially, while lymphangiogenesis contributes to metastatic dissemination. Given this double function in angiogenesis and lymphangiogenesis, endothelial OPA1 deletion not only curtails tumor growth but also metastasis dissemination. In a proof of principle experiment, we identified a specific OPA1 inhibitor, N-(1,5-dimethyl-3-oxo-2-phenyl-2,3-dihydro-1H-pyrazol-4-YL)-3-methyl-1-PH+ (MYLS22) that curtails tumor growth by targeting endothelial OPA1 [5]. The remarkable finding that a small molecule inhibitor of a key mitochondria-shaping protein can blunt tumor growth by inhibiting angiogenesis paves the way to develop more refined, second generation chemical OPA1 inhibitors as antiangiogenesis drugs. The ability to inhibit angiogenesis at a step different than VEGF receptor activation might also offer a new therapeutic perspective for cancers that develop anti-VEGF resistance.

**Figure 1:**
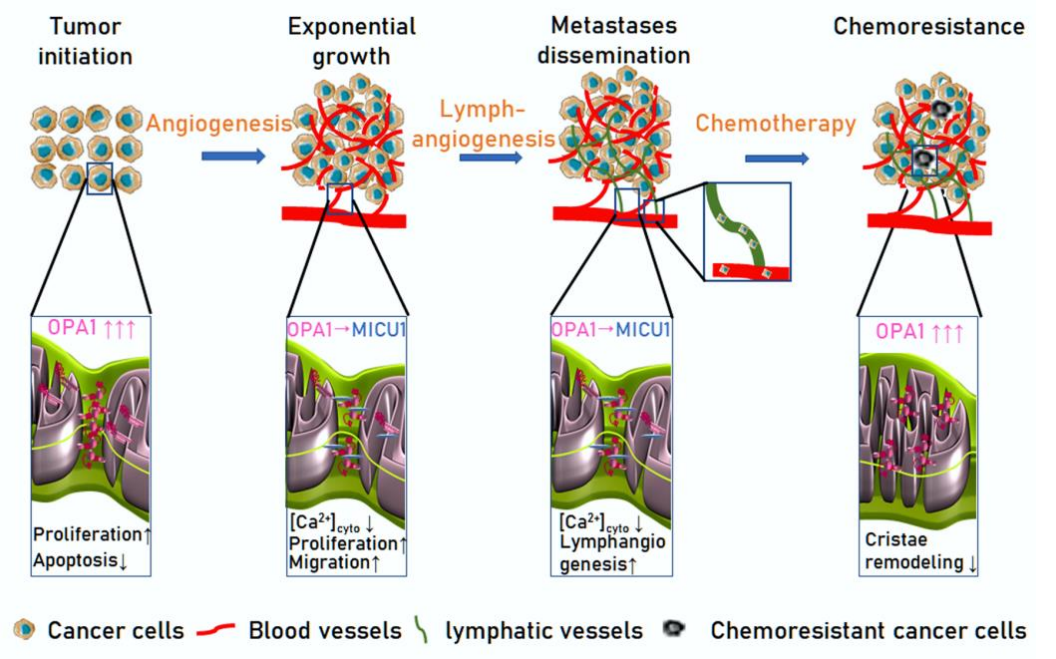
**OPA1 participates in different steps of tumorigenesis and metastatization**. Tumor initiation: OPA1 is upregulated in a variety of cancer types, favoring proliferation, and protecting them from apoptosis. Exponential growth: endothelial OPA1 enhances tumor vascularization. By interacting with the mitochondrial calcium uptake machinery component MICU1, OPA1 limits Ca^2+^ accumulation in the cytosol, a negative regulator endothelial cells proliferation and migration. (3) Metastases dissemination: Endothelial OPA1 favors lymphatic vessels differentiation from venous blood vessels. (4) Chemoresistance: OPA1 overexpression in cancer cells curtails cristae remodeling, a key step for cytochrome c release and induction of cell death by targeted and conventional anti-cancer drugs.

OPA1 is also emerging as a key molecule for cancer cell biology and resistance to therapy. OPA1 was identified as one of the 8 novel prognosis-related genes that show a copy number gain (CNG) in several cancers. Indeed, OPA1 is significantly overexpressed in stomach, lung, ovarian, esophageal, cervical, pancreatic and breast cancer. Pancreatic ductal adenocarcinoma (PDA) and triple negative breast cancer (TNBC) are among the deadliest malignancies. Both represent a major clinical challenge due to the lack of reliable prognostic markers and targeted therapies. Interestingly, in PDA higher OPA1 levels are associated with a worse prognosis [6]. These results further suggest that targeting OPA1 might curtail not only angiogenesis as we discovered, but also directly cancer cells. As a proof of principle, we cite the example of acute myeloid leukemia (AML). In an unbiased screening for genes conferring resistance to Venetoclax, an FDA approved Bcl-2 antagonist used in the treatment of AML, OPA1 was found to be upregulated and its deletion reverted Venetoclax resistance [7]. Further studies are required to understand whether chemical OPA1 inhibition can be beneficial in the case of Venetoclax-resistant AML and more in general to overcome resistance to targeted and conventional tumor therapy. Overall, we can conclude that the mitochondrial protein OPA1 is crucial for different key process dysregulated in cancer: apoptosis and cellular proliferation by its direct control of mitochondrial biology; tumor growth and metastatization by licensing angiogenesis; and resistance to targeted therapy at least in AML (Figure 1): Specific, effective, safe OPA1 inhibitors like the ones being developed in our laboratory would allow addressing whether anti-OPA1 molecules can be added to the shelf of cancer therapeutics.
